# *PAX6 *mutations: genotype-phenotype correlations

**DOI:** 10.1186/1471-2156-6-27

**Published:** 2005-05-26

**Authors:** Ioanna Tzoulaki, Ian MS White, Isabel M Hanson

**Affiliations:** 1 School of Molecular and Clinical Medicine, University of Edinburgh, Molecular Medicine Centre, Western General Hospital, Crewe Road, Edinburgh, EH4 2XU, UK; 2 School of Biological Sciences, Institute of Evolutionary Biology, Ashworth Laboratories, West Mains Road, Edinburgh, EH9 3JT, UK

## Abstract

**Background:**

The PAX6 protein is a highly conserved transcriptional regulator that is important for normal ocular and neural development. In humans, heterozygous mutations of the *PAX6 *gene cause aniridia (absence of the iris) and related developmental eye diseases. *PAX6 *mutations are archived in the Human PAX6 Allelic Variant Database, which currently contains 309 records, 286 of which are mutations in patients with eye malformations.

**Results:**

We examined the records in the Human PAX6 Allelic Variant Database and documented the frequency of different mutation types, the phenotypes associated with different mutation types, the contribution of CpG transitions to the *PAX6 *mutation spectrum, and the distribution of chain-terminating mutations in the open reading frame. Mutations that introduce a premature termination codon into the open reading frame are predominantly associated with aniridia; in contrast, non-aniridia phenotypes are typically associated with missense mutations. Four CpG dinucleotides in exons 8, 9, 10 and 11 are major mutation hotspots, and transitions at these CpG's account for over half of all nonsense mutations in the database. Truncating mutations are distributed throughout the *PAX6 *coding region, except for the last half of exon 12 and the coding part of exon 13, where they are completely absent. The absence of truncating mutations in the 3' part of the coding region is statistically significant and is consistent with the idea that nonsense-mediated decay acts on *PAX6 *mutant alleles.

**Conclusion:**

The PAX6 Allelic Variant Database is a valuable resource for studying genotype-phenotype correlations. The consistent association of truncating mutations with the aniridia phenotype, and the distribution of truncating mutations in the *PAX6 *open reading frame, suggests that nonsense-mediated decay acts on *PAX6 *mutant alleles.

## Background

The *PAX6 *gene was cloned during the search for genes underlying the WAGR syndrome (Wilms tumor, aniridia, genitourinary abnormalities and mental retardation; MIM 194072) [1]. WAGR syndrome is caused by hemizygous deletions of 11p13 that remove one copy of *PAX6 *and one copy of *WT1 *[1,2]. Intragenic *PAX6 *mutations were subsequently identified in numerous non-syndromic aniridia patients, confirming *PAX6 *as the aniridia gene (MIM 106210) [3-6]. Studies on WAGR patients and aniridia patients with chromosomal rearrangements clearly demonstrated that aniridia could be caused by deletion of one copy of the *PAX6 *gene [1,2]. Thus it was proposed that aniridia results from *PAX6 *haploinsufficiency and is caused by loss-of-function of one allele [1,5-7].

**Figure 1:**
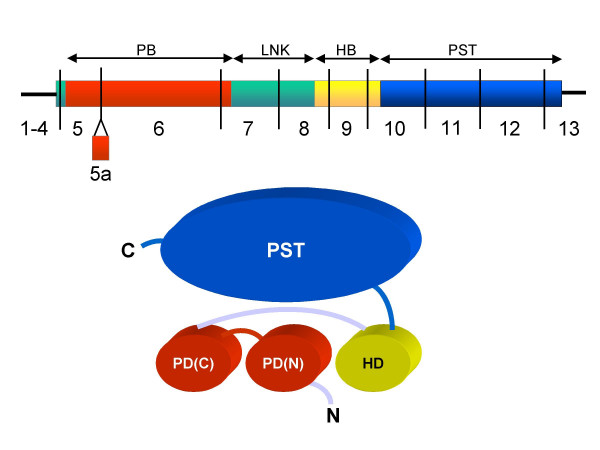
The PAX6 cDNA and protein. Top: the *PAX6 *cDNA is represented as a horizontal rectangle with the different coding regions indicated: PB, paired box, LNK, linker region, HB, homeobox, PST, proline/serine/threonine-rich region. Exon boundaries are indicated by vertical black lines. 5a is alternatively spliced exon in the paired box. Thick horizontal lines indicate untranslated regions (not to scale). Bottom: cartoon of the PAX6 protein showing the different functional domains. N, N-terminus of protein; C, C-terminus; PB(N), N-terminal subdomain of paired domain; PB(C), C-terminal subdomain of paired domain; HD, homeodomain; PST, PSTdomain.

The *PAX6 *gene encodes a highly conserved transcriptional regulatory protein that is expressed in the developing eye, brain, spinal cord and pancreas [8]. The 5' two-thirds of the open reading frame (ORF) encode two DNA binding domains, a paired domain and a homeodomain (Figure 1) [9,10]. The DNA binding domains are separated by a 79-amino acid linker peptide. The 3' third of the ORF encodes a proline, serine and threonine-rich (PST) domain that has transcriptional trans-activation function [11] (Figure 1). The last 40 amino acids of the PST domain constitute a highly conserved C-terminal peptide that has been implicated in modulation of DNA binding by the homeodomain [12].

PAX6 mutations are archived in the Human PAX6 Allelic Variant Database [13,14]. The database now contains 309 records, each reporting independently ascertained sequence variations in the PAX6 gene. Two hundred and eighty-six of these are associated with pathological mutations that cause congenital eye malformations. The most common of these malformations is aniridia, which is chiefly characterised by congenital absence of the iris, but which also affects the cornea, lens and retina. *PAX6 *mutations also cause a range of non-aniridia phenotypes such as optic nerve defects, keratitis, microphthalmia, and foveal hypoplasia [15-17]. This database records allow analysis of the distribution of mutations in the gene and the relationship between genotype and phenotype.

Although a comprehensive review of the mutations in the database has been published previously [18] we wanted to re-analyse the data for two reasons. First, the last review was published seven years ago and the number of records has greatly increased, from 87 to 309. Second, it is instructive to consider the effect of emerging molecular mechanisms that act on mutant alleles, such as nonsense-mediated decay. Nonsense-mediated decay (NMD) is the process by which mRNAs that contain premature termination codons (PTCs) are degraded before they produce large quantities of truncated proteins [19,20]. NMD is of relevance to the *PAX6 *mutation spectrum because mutations that introduce a PTC into the *PAX6 *open reading frame are very common [18]. Before the discovery of NMD it was widely thought that PTC-containing mutant alleles generated truncated proteins [5,6]. Some researchers speculated that PAX6 proteins truncated after the homeodomain might have dominant negative activity [21-23]. The two intact DNA binding domains, divorced from the normal trans-activation domain, could theoretically bind to target DNA sequences without activating downstream genes and could potentially interfere with the function of the normal PAX6 protein. A variety of experimental assays showed that PAX6 proteins with C-terminal deletions do indeed have potent dominant negative activity [21,22,24]. It might be expected therefore that truncating mutations after the homeodomain could potentially lead to phenotypes more severe than, or markedly different from, truncating mutations in the DNA binding domains. Alternatively, if nonsense-mediated decay acts on PTC mutations *in vivo*, all PTC alleles will effectively be null alleles, and no phenotypic difference should be observed. This idea can be explored by examining the records in the PAX6 Allelic Variant Database

In this paper we review the mutations archived in the PAX6 Allelic Variant Database. We show that over three-quarters of aniridia cases are caused by mutations that introduce a PTC into the *PAX6 *open reading frame. In contrast, most non-aniridia phenotypes are associated with missense mutations. We also show that four CpG dinucleotides are major mutational hotspots, and account for half of all nonsense mutations in the database. Finally we attempt to reconcile the observed *PAX6 *mutation spectrum with the work done on truncated PAX6 proteins. We suggest that the *PAX6 *mutation spectrum is consistent with the idea that nonsense-mediated decay is a major mechanism acting on *PAX6 *mutant alleles, and consequently that most truncated proteins are unlikely to be produced at significant levels *in vivo*. Among the existing records, there are no truncating mutations in the 3' part of the coding region where RNA surveillance would not be predicted to act. This suggests that 3' mutations do in fact yield dominant negative alleles that may cause severe phenotypes, but these have not yet been ascertained.

## Results and discussion

### Truncating mutations in the *PAX6 *gene are predominantly associated with aniridia

The PAX6 Allelic Variant Database contains 309 records of which 286 refer to pathological mutations in the *PAX6 *coding region (exons 4–13, Figure 1) or the consensus splice sites directly flanking the coding exons. The remaining records describe polymorphisms.

Each of the 286 disease-associated mutations was classified into one of six categories according to the apparent effect of each genomic change. The six categories are nonsense mutations, splicing mutations, frame-shifting insertions or deletions, in-frame insertions or deletions, missense mutations and run-on mutations. Details of these are given in Table 1.

**Table 1:** Categories of mutation in the PAX6 Allelic Variant Database

Category	Definition
Nonsense	Single nucleotide substitution creates a stop codon in the open reading frame.
Splicing	Nucleotide substitution, deletion or insertion in consensus splice site.
Frame-shifting insertion or deletion	Deletion or insertion of nucleotides in the open reading frame – total number not divisible by three.
In-frame insertion or deletion	Deletion or insertion of nucleotides in the open reading frame – total number divisible by three.
Missense	Single nucleotide substitution changes one amino acid codon to another in the open reading frame.
Run-on	Nucleotide substitution, insertion or deletion changes the termination codon to an amino acid codon. Translation is predicted to continue into the 3'UTR.

Each of the 286 disease-associated mutations in the database was assigned to one of these categories. Twelve compound mutations (each involving more than one mutational event) were categorised according to the final consequence of the mutation (see Methods). 3'UTR, 3' untranslated region.

**Figure 2:**
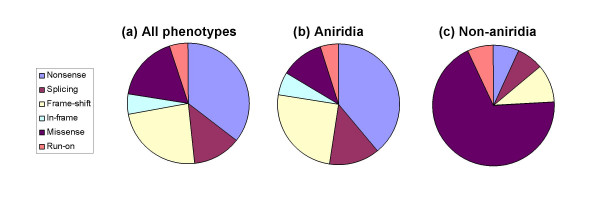
Distribution of different mutation types in the PAX6 Allelic Variant Database. (a) All disease-associated mutations in the database; (b) mutations causing aniridia; (c) mutations causing non-aniridia phenotypes. Mutation definitions are given in Table 1.

The exon-by-exon distribution of mutation type for 286 pathological mutations is shown in Table 2. Of the 286 mutations, 102 (35.7%) are nonsense mutations, 36 (12.6%) are splice mutations, 68 (23.8%) are frame-shifting insertions or deletions, 16 (5.6%) are in-frame insertions or deletions, 50 (17.5%) are missense mutations and 14 (4.8%) are run-on mutations (Figure 2a).

**Table 2:** Exon-by-exon distribution of 286 disease-associated mutations in the PAX6 Allelic Variant Database

	**4**	**5**	**5a**	**6**	**7**	**8**	**9**	**10**	**11**	**12**	**13**	
**Mutation type**												**Total**

N	0	6	0	9	7	24	23	14	16	3	0	**102**
S	2	4	0	4	1	1	4	6	5	9	0	**36**
FS	0	14	0	19	11	4	4	6	1	6	3	**68**
IF	0	3	0	13	0	0	0	0	0	0	0	**16**
M	3	13	5	14	3	2	1	1	0	6	2	**50**
RO	0	0	0	0	0	0	0	0	0	0	14	**14**
**Total**	**5**	**40**	**5**	**59**	**22**	**31**	**32**	**27**	**22**	**24**	**19**	**286**

N, nonsense mutation; S, splicing mutation; FS, frame-shifting insertion or deletion; IF, in-frame insertion or deletion; M, missense mutation; RO, run-on mutation. For a full definition of mutation types, see Table 1.

Nonsense mutations, splice mutations and frame-shifting insertions or deletions typically result in the introduction of a PTC into the open reading frame. In the PAX6 database, these three categories account for 72% of all disease-associated mutations.

Of the 286 pathological mutations, 257 (89.9%) are associated with aniridia and 29 (10.1%) are associated with other phenotypes, including isolated foveal hypoplasia, microphthalmia and optic nerve defects.

The exon-by-exon distribution of mutation type for 257 aniridia-associated mutations is shown in Table 3. Of the 257 mutations, 100 (38.9%) are nonsense mutations, 34 (13.2%) are splice mutations, 65 (25.3%) are frame-shifting insertions or deletions, 16 (6.2%) are in-frame insertions or deletions, 30 (11.7%) are missense mutations and 12 (4.7%) are run-on mutations (Figure 2b). The proportion of missense mutations has decreased from 17.5% of all cases, to 11.7% of aniridia cases while mutations that introduce a PTC (nonsense, splicing and frame-shifting mutations) have increased from 72% of all cases to 77% of aniridia cases.

**Table 3:** Exon-by-exon distribution of 257 mutations that cause aniridia.

**Exon**	**4**	**5**	**5a**	**6**	**7**	**8**	**9**	**10**	**11**	**12**	**13**	
**Mutation type**												**Total**

N	0	6	0	9	7	23	23	14	16	2	0	**100**
S	1	4	0	4	1	1	4	6	4	9	0	**34**
FS	0	13	0	19	11	4	4	6	1	5	2	**65**
IF	0	3	0	13	0	0	0	0	0	0	0	**16**
M	3	11	0	10	0	2	1	0	0	2	1	**30**
RO	0	0	0	0	0	0	0	0	0	0	12	**12**
**Total**	**4**	**37**	**0**	**55**	**19**	**30**	**32**	**26**	**21**	**18**	**15**	**257**

N, nonsense mutation; S, splicing mutation; FS, frame-shifting insertion or deletion; IF, in-frame insertion or deletion; M, missense mutation; RO, run-on mutation. For a full definition of mutation types, see Table 1.

The exon-by-exon distribution of mutation type for 29 mutations in non-aniridia cases is shown in Table 4. Of the 29 mutations, 2 (6.9%) are nonsense mutations, 2 (6.9%) are splice mutations, 3 (10.3%) are frame-shifting insertions or deletions, 20 (69%) are missense mutations and 2 (6.9%) are run-on mutations (Figure 2c). Missense mutations account for over two-thirds of non-aniridia phenotypes, while mutations that introduce a PTC are much less common than in the database as a whole, accounting for just 7 of the cases (24%).

**Table 4:** Exon-by-exon distribution of 29 mutations that cause phenotypes other than aniridia.

**Exon**	**4**	**5**	**5a**	**6**	**7**	**8**	**9**	**10**	**11**	**12**	**13**	
**Mutation type**												**Total**

N	0	0	0	0	0	1	0	0	0	1	0	**2**
S	1	0	0	0	0	0	0	0	1	0	0	**2**
FS	0	1	0	0	0	0	0	0	0	1	1	**3**
IF	0	0	0	0	0	0	0	0	0	0	0	**0**
M	0	2	5	4	3	0	0	1	0	4	1	**20**
RO	0	0	0	0	0	0	0	0	0	0	2	**2**
**Total**	**1**	**3**	**5**	**4**	**3**	**1**	**0**	**1**	**1**	**6**	**4**	**29**

N, nonsense mutation; S, splicing mutation; FS, frame-shifting insertion or deletion; IF, in-frame insertion or deletion; M, missense mutation; RO, run-on mutation. For a full definition of mutation types, see Table 1.

This analysis shows that the aniridia phenotype is predominantly associated with mutations that introduce a PTC, while non-aniridia phenotypes are predominantly associated with missense mutations. The missense mutations that cause non-aniridia phenotypes may do so by generating hypomorphic proteins that are able to carry out some but not all of the normal functions of PAX6, such as the correct regulation of downstream target genes [15,28]. The missense mutations that cause non-aniridia phenotypes are predominantly located in the paired domain (exons 5, 5a, 6 and 7, Table 4), suggesting that partially impaired DNA binding may be a major mechanism by which variant phenotypes arise [10,28,29]. Missense mutations can cause full-blown aniridia (Table 3), presumably by creating PAX6 proteins with little or no function [29]. Missense mutations associated with non-aniridia phenotypes typically affect a subset of the ocular tissues involved in full aniridia, such as the fovea, the optic nerve or the iris [15,29].

### Mutation hotspots in the *PAX6 *coding region

Nonsense mutations are the single most common mutation type in aniridia patients (and in the whole database) while missense mutations are the most common cause of other phenotypes (Figure 2). Both nonsense and missense mutations are caused by single nucleotide substitutions. To learn more about how these mutations might arise, we focussed on the distribution of CpG dinucleotides in the *PAX6 *open reading frame, since CpG transitions are the most common single nucleotide substitutions in the human genome [30].

**Figure 3:**
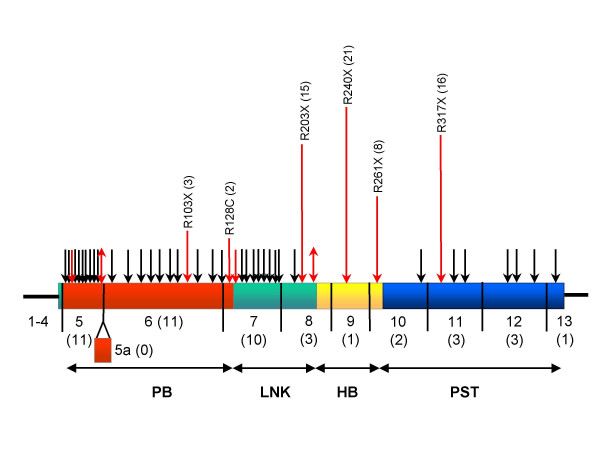
Distribution of CpG dinucleotides in the *PAX6 *open reading frame. The *PAX6 *cDNA is represented as a horizontal rectangle with the different coding regions indicated: PB, paired box, LNK, linker region, HB, homeobox, PST, proline/serine/threonine-rich region. Exon boundaries are indicated by vertical black lines. Exon numbers are shown beneath the cDNA, with the number of CpG's in brackets. Above the cDNA, each of the forty-five CpG dinucleotides in the PAX6 ORF is indicated by an arrow. Red arrows indicate the ten CpG's at which a nucleotide transition has occurred. Single-headed arrows indicate that the CpG deamination has occurred only on the forward strand (CpG > TpG). Double-headed arrows indicate that deamination has occurred both on the forward strand (CpG > TpG) and the reverse strand (CpG > CpA). Elongated red arrows indicate those CpG's that have been hit more than once on the forward strand; the resultant mutation is shown together with the number (in brackets) of independent records in the database.

There are 45 CpG dinucleotides in the *PAX6 *coding region (Figure 3). CpG deamination can give rise to two new dinucleotides, TpG and CpA, depending on whether the C>T conversion takes place on the forward strand or the reverse strand. In the existing records, transitions at 10 of the 45 CpG's have been reported (Figure 3, Table 5). Eight CpG's have been mutated on one strand only, while two have been mutated on both strands (Figure 3, Table 5). Of the twelve changes, six cause nonsense mutations, five cause missense mutations and one causes a synonymous (neutral) substitution (Table 5).

**Table 5:** CpG transitions in the *PAX6 *open reading frame.

Nucleotide change	Codon change	Location	Mutation type	Reports
414G>A	ACG>ACA (G18R)	PD exon 5	Missense	1
494C>T	CGA>TGA (R44X)	PD exon 5	Nonsense	1
493G>A	CGA>CAA (R44Q)	PD exon 5	Missense	1
669C>T	CGA>TGA (R103X)	PD exon 6	Nonsense	3
744C>T	CGC>TGC (R128C)	PD exon 7	Missense	2
782C>T	GAC>GAT (D140D)	LNK exon 7	Neutral	1
969C>T	CGA>TGA (R203X)	LNK exon 8	Nonsense	15
984C>T	CGG>TGG (R208W)	LNK exon 8	Missense	1
985G>A	CGG>CAG (R208Q)	LNK exon 8	Missense	1
1080C>T	CGA>TGA (R240X)	HD exon 9	Nonsense	21
1143C>T	CGA>TGA (R261X)	HD exon 10	Nonsense	8
1311C>T	CGA>TGA (R317X)	PST exon 11	Nonsense	16

PD, paired domain; LNK, linker region; HD, homeodomain; PST, proline/serine/threonine-rich region. 'Reports' indicates how many times the mutation has been reported in the PAX6 Allelic Variant Database.

Sense-strand deamination of CpG in an arginine codon CGA creates a termination codon TGA. The *PAX6 *ORF contains six CGA codons and all of these have been 'hit' at least once to give nonsense mutations (Table 5). It is noticeable that four CpG's in CGA codons in exons 8, 9, 10 and 11 (R203X, R240X, R261X and R317X) are a major source of nonsense mutations (Table 5, Figure 3). Together these four CpG's have been hit 60 times. These hits all cause aniridia and account for 21% of all mutations in the database and 59% of all nonsense mutations.

The observation that CpG's in exons 8, 9, 10 and 11 are a major source of mutations can be explained at least in part by the nucleotide composition and methylation status of the genomic *PAX6 *gene. The 5' two-thirds of the gene (from the promoter up to and including exon 7) are part of an unusually large CpG island. This region of the gene is very GC-rich and has a high frequency of CpG dinucleotides, most of which are unmethylated [31]. The last third of the gene, containing exons 8–13, is more similar to bulk genomic DNA, with a lower GC content. The frequency of CpG dinucleotides is low, but those that exist tend to be methylated, and methylation greatly increases the frequency of spontaneous deamination of cytosine, resulting in C>T transition [30,31]. Although only 13 of the 45 CpG's are in exons 8–13 (Figure 3), these are in the methylated region of the gene and are therefore much more likely to be 'hit'.

### C-terminal truncating mutations are not associated with more severe phenotypes

When the first *PAX6 *mutations were discovered in aniridia patients, it quickly became apparent that mutations that introduce a PTC into the open reading frame are common [3-6]. Speculation arose that mutations causing translational termination after the homeodomain might yield dominant negative forms of the PAX6 protein, because truncated proteins containing only the DNA binding domains could theoretically bind to target DNA sequences without activating downstream genes and hence interfere with the function of the normal PAX6 protein [21-23]. A variety of studies then demonstrated that PAX6 proteins with C-terminal deletions do indeed have dominant negative activity [21,22,24]. It might therefore be expected that individuals with truncating mutations in the PST domain would have very low levels of normal PAX6 activity and this could result in a phenotype more severe than, or markedly different from, individuals with truncating mutations before the PST domain.

Mutations that introduce a PTC – nonsense mutations, splicing mutations and frame-shifting insertions and deletions – are scattered throughout the *PAX6 *open reading frame (Table 2). We examined the database records for evidence that late-terminating mutations (in the PST domain) cause different phenotypes.

Of 151 mutations that introduce a PTC before the PST domain (ie in the paired box, the linker region or the homeobox), 150 are associated with aniridia or closely related variants such as partial aniridia and iris hypoplasia. The remaining mutation is associated with optic nerve hypoplasia [15].

Of 43 mutations that introduce a PTC into the PST domain, 41 are associated with aniridia or closely related phenotypic variants. The remaining two mutations are associated with keratitis [16] and congenital cataracts [11], phenotypes that clearly overlap with aniridia. Therefore there is no evidence from the existing records that truncating mutations in the PST domain are associated with more severe phenotypes. Rather the data suggest that truncating mutations are overwhelmingly associated with aniridia regardless of their location in the gene.

How can the uniformity of patient phenotypes be reconciled with experimental data demonstrating dominant negative effects? One explanation is that the dominant negative tests may not be physiologically relevant. The experiments were performed on intronless cDNA constructs that terminate at an engineered stop codon and are designed to produce large quantities of the truncated protein. In contrast the patients have PTCs that occur in the context of an intact *PAX6 *gene. Once transcribed, PTC-containing RNAs are likely to be degraded by nonsense-mediated decay, a universal mechanism for preventing the accumulation of truncated proteins [19,20]. Nonsense mediated decay is inextricably linked to the synthesis, processing, splicing and preliminary translation of mRNAs derived from genomic genes [19,20]. Experimental cDNA constructs bypass these mechanisms to direct the synthesis of high levels of proteins that would not normally be made in the cell [19].

We propose that the dominant negative tests do not accurately reflect the *in vivo *consequence of truncating mutations. The simplest interpretation of the phenotypic data is that nonsense-mediated decay acts on most PTC-containing RNAs to generate null alleles.

The existing data are entirely consistent with the hypothesis that aniridia is a true haploinsufficiency phenotype, caused by loss of function of one allele, either by deletion or intragenic mutation. As mentioned previously, 77% of all aniridia-associated mutations in the database result in the introduction of a PTC. Therefore nonsense-mediated decay may be the major mechanism by which *PAX6 *null alleles are generated.

### Absence of nonsense mutations at the 3' end of the *PAX6 *coding region

**Figure 4:**
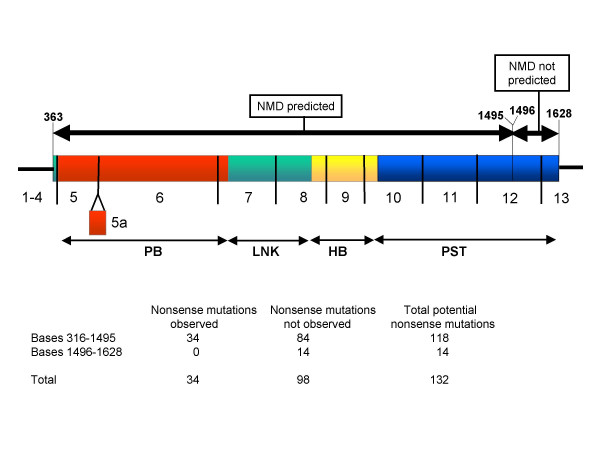
Absence of nonsense mutations at the 3' end of the *PAX6 *coding region. The *PAX6 *open reading frame is represented as a horizontal rectangle; the untranslated regions are shown as thick black lines (not to scale). Exon boundaries are shown as vertical lines. PB, paired box; LNK, linker region; HB, homeobox; PST, PST region. Above the cDNA, thick double-headed arrows divide the coding region into two parts. Between bases 363–1495, nonsense-mediated decay is predicted to act on truncating mutations. The region from bases 1496–1628 is predicted to escape nonsense-mediated decay. The number of potential and observed nonsense mutations in the different zones of the coding region is shown in the lower part of the figure. No nonsense mutations have been observed in the region that escapes NMD, even though 14 codons could potentially give rise to nonsense mutations.

Any hypothesis concerning the role of nonsense-mediated decay must take into account the observation that it does not act on the 3' extreme of a coding region. The surveillance mechanism uses intron/exon boundaries as cues for detection of PTCs and typically does not operate on the last exon, or the last 50 bases of the penultimate exon [19,32]. In the *PAX6 *gene, the zone that would escape NMD would encompass the last 50 bp of exon 12 (from base 1496 onwards) and the first 83 bases of exon 13 up to the normal stop codon. This corresponds to the last 44 codons of the open reading frame (Figure 4). Truncating mutations in this region of the *PAX6 *gene should not be acted on by NMD and could theoretically generate dominant negative forms of the protein. In experimental assays, even a short reduction of 37 amino acids gave potent dominant negative effects [21].

We inspected the database records to see what kinds of mutations are present in the region that is predicted to escape NMD (base 1496 onwards). Strikingly there are no nonsense mutations in this region (Table 6). There are four splicing mutations and five frame-shifting deletions, but the predicted consequence of all of these is run-on translation into the 3'UTR rather than introduction of a PTC [5,14,33,35,36]. This is in sharp contrast to the 5' part of exon 12 (up to base 1495), which contains a variety of nonsense and frame-shifting mutations, all of which are predicted to introduce a PTC (Table 6).

**Table 6:** Outcome of potential PTC-creating mutation in exons 12 and 13.

Mutation	Location	Consequence of mutation	Type	NMD predicted
1399delC	Exon 12	PTC at TGA starting at base 1453	FSD	Yes
1410delC	Exon 12	PTC at TGA starting at base 1453	FSD	Yes
1420C>G (S353X)	Exon 12	Immediate PTC	N	Yes
1424C>G (Y354X)	Exon 12	Immediate PTC	N	Yes
1426ins5	Exon 12	PTC at TGA starting at base 1469	FSI	Yes
1452delG	Exon 12	PTC at TGA starting at base 1453	FSD	Yes
1469T>G (Y369X)	Exon 12	Immediate PTC	N	Yes
1513del4	Exon 12	Run-on into 3'UTR	FSD	No
1520delGGinsT	Exon 12	Run-on into 3'UTR	FSD	No
1545G>C	Exon 12	Run-on into 3'UTR	S	No
IVS12+1del3	Intron 12	Run-on into 3'UTR	S	No
IVS12+4A>G	Intron 12	Run-on into 3'UTR	S	No
IVS12+5G>A	Intron 12	Run-on into 3'UTR	S	No
1562delT	Exon 13	Run-on into 3'UTR	FSD	No
1601delT	Exon 13	Run-on into 3'UTR	FSD	No
1615del10	Exon 13	Run-on into 3'UTR	FSD	No

This table shows the outcome of every known *PAX6 *mutation from the start of exon 12 onwards that could potentially introduce a premature termination codon. Mutations up to and including nucleotide 1495 are within the region in which nonsense-mediated decay is predicted to occur, while mutations from nucleotide 1496 onwards are predicted to escape nonsense-mediated decay. IVS, intervening sequence (intron); FSD, frame-shifting deletion; FSI, frame shifting insertion; N, nonsense mutation; S, splice mutation. PTC, premature termination codon; 3' UTR, 3' untranslated region.

Thus there appears to be an absence of truncating mutations in the part of the gene that is predicted to escape NMD. To investigate this further, we looked at the distribution of potential nonsense codons in the *PAX6 *coding region. The *PAX6 *open reading frame contains 132 codons that could be mutated to stop codons by a single base change. 102 nonsense mutations have been observed in patients and these occur at 34 of the possible 132 sites (Figure 4).

Fourteen of the potential nonsense codons lie within the region predicted to escape NMD (base 1496 onwards) but none of the resultant mutations has been observed to date (Figure 4). Assuming a random distribution of all unique nonsense mutations along the potential sites at which a nonsense mutation could occur, the probability of observing zero mutations in the non-surveillance zone is 0.012 calculated using Fisher's exact test (Figure 4). Thus the absence of mutations in exon 13 and the last 50 bp of exon 12 is unlikely to have arisen by chance.

As mentioned above, 9 different frame-shifting and splice mutations have been reported in the region predicted to escape NMD but none of these introduces a PTC. Rather they are all predicted to cause run-on translation into the 3' untranslated region (Table 6) [5,7,14,33-37].

The phenotypes associated with run-on mutations are well documented because the database contains details of 14 run-on mutations in which the normal termination codon is altered to a coding codon. All of these patients have aniridia or ocular defects within the aniridia spectrum such as iris hypoplasia, foveal hypoplasia, cataracts and nystagmus.

Thus translation beyond the normal stop codon is consistently associated with an aniridia-like phenotype, which suggests that run-on mutations generate simple loss-of-function alleles. Nonsense-mediated decay would not be predicted to act on such alleles because there is no PTC; therefore the proposed loss of function may result from the addition of an extra peptide at the C-terminal end of the PAX6 protein. The C-terminus of PAX6 is highly conserved and appears to play a role in the stabilisation of DNA binding by the homeodomain [12], so any disruption to the structure of the C-terminal region could have profound effects on the function of the PAX6 protein. It should however be emphasised that the function of run-on PAX6 proteins has not yet been tested; therefore confirmation of the hypothesis that run-on proteins show loss of function rather than dominant negative activity awaits further experimentation.

The known *PAX6 *mutation spectrum is devoid of mutations that introduce a PTC into the non-surveillance zone. Such mutations must surely arise, yet clearly they are not associated with aniridia. Given the evidence that even short truncations of the PAX6 protein cause strong dominant negative effects [21], we propose that termination mutations in the last part of the gene cause phenotypes significantly more severe than aniridia. These phenotypes may resemble that of the only confirmed case of an individual with a lethal compound heterozygous *PAX6 *mutation and may include anophthalmia, arhinia and severe central nervous system defects [11].

## Conclusion

We have reviewed the mutations in the PAX6 Allelic Variant Database. Aniridia is typically caused by mutations that introduce a PTC, while non-aniridia phenotypes are cause by missense mutations. Transitions at four CpG dinucleotides in the methylated part of the gene make a major contribution to the burden of *PAX6 *nonsense mutations.

We have reasoned that nonsense-mediated decay acts on *PAX6 *mutant alleles. Mutations that introduce a PTC are consistently associated with aniridia or closely related phenotypes, regardless of where they occur in the gene. There is a statistically significant absence among the existing records of nonsense mutations in exon 13 and the last 50 bp of exon 12, where NMD would not be predicted to act.

Mutations that introduce a termination codon before the last 50 bases of exon 12 are likely to be acted on by nonsense-mediated decay and are probably functionally null. However mutations that introduce a termination codon within the last 50 bases of exon 12, or within exon 13, are likely to generate proteins with significant dominant negative activity. These mutations are not associated with aniridia and may cause very severe phenotypes that have not yet been ascertained. The effect of NMD on phenotypic severity has been experimentally demonstrated for *SOX10 *and *MPZ*, mutations of which cause neurochristopathies and myelinopathies respectively [38]. In *SOX10 *and *MPZ*, truncating mutations at the 3' end of the open reading frame escape NMD and generate dominant negative proteins that cause much more severe phenotypes than more 5' mutations [38].

The mutation spectrum of a gene can yield important insights into the molecular mechanisms that act on mutant alleles and the phenotypes that are likely to be associated with mutations in that gene.

## Methods

### *PAX6 *cDNA reference sequence and numbering

The *PAX6 *cDNA sequence used in this paper is taken from the PAX6 Allelic Variant Database [25]. The coding region runs from base 363 (in exon 4) to base 1628 (in exon 13). The PST region extends from base 1169 to base 1628.

### *PAX6 *mutations and phenotypes

Data on the *PAX6 *mutation spectrum were collected from the PAX6 Allelic Variant Database. Only pathological mutations were considered, either within the coding region of the gene (bases 363–1628) or within the consensus splice acceptor and donor sequences of introns. Coding region polymorphisms and intronic changes outside the splice consensus sequences were not considered. Each mutation was placed into one of six categories (nonsense, splicing, frame-shifting insertion/deletion, in-frame insertion/deletion, missense and run-on – see Table 1 for definitions). There are twelve compound mutations in the database, each apparently involving more than one mutational event, such as an insertion and a deletion. These were categorised according to the final consequence of the mutation. For example the compound mutation 495delAinsCAT has a net effect of inserting two bases and is therefore categorised as a frame-shifting mutation.

### Distribution of CpG dinucleotides and potential termination codons

The Mutability program determines the number and distribution of CpG dinucleotides and the number and distribution of potential nonsense and missense mutations arising from single nucleotide substitutions in a cDNA sequence. It is freely available through the PAX6 Allelic Variant Database web site [26]. We used the Mutability program to determine the location of CpG dinucleotides in the PAX6 open reading frame, and to determine the total number of codons that could potentially be mutated to a stop codon by a single nucleotide change [26]. The analysis was carried out on the complete coding region of *PAX6*, including exon 5a. For exons 4 and 13, which contain the initiation and termination codons respectively, only the coding DNA was considered.

### Fisher's exact test

The number of observed nonsense mutations in the coding region was obtained from the PAX6 Allelic Variant Database. The number of nonsense mutations that were not observed was calculated by subtracting the number of observed mutations from the number of potential mutations (calculated using the Mutability program, above). The 'non-surveillance zone' of the coding region, in which RNA surveillance should not act was defined as the coding region of exon 13 and the last 50 bp of exon 12, ie from nucleotide c.1496 onwards. Application of Fisher's exact test to the 2 × 2 table shown in Figure 4 [34, 84, 0, 1] gives a one-tailed probability of p = 0.012, which is significant beyond the 5% level and allows rejection of the null hypothesis that the observed nonsense mutations are randomly distributed throughout the coding region. The Fisher's exact test calculation was carried out using the tool at [27]

## Abbreviations

NMD, nonsense-mediated decay; ORF, open reading frame; PTC; premature termination codon.

## Authors' contributions

IT carried out the bioinformatic analyses. IMSW advised on and verified the statistical analysis. IMH conceived of and supervised the study, and prepared the manuscript. All authors read and approved the final version.
